# An Up-To-Date Investigation Into the Correlation Between Proton Pump Inhibitor Use and the Clinical Efficacy of Immune Checkpoint Inhibitors in Advanced Solid Cancers: A Systematic Review and Meta-Analysis

**DOI:** 10.3389/fonc.2022.753234

**Published:** 2022-02-24

**Authors:** Chaoxing Liu, Huaijuan Guo, Haiyan Mao, Jiandong Tong, Mengxue Yang, Xuebing Yan

**Affiliations:** ^1^ Department of Oncology, The Affiliated Hospital of Yangzhou University, Yangzhou University, Yangzhou, China; ^2^ Department of Oncology, Hefei Cancer Hospital, Chinese Academy of Sciences, Heifei, China

**Keywords:** immune checkpoint inhibitors, proton pump inhibitors, meta-analysis, prognosis, cancer

## Abstract

**Background:**

Although immune checkpoint inhibitors (ICIs) have revolutionized the current anticancer therapies, a considerable proportion of patients are found to hardly benefit from these drugs. Accumulating studies have demonstrated that concomitant proton pump inhibitor (PPI) use may affect the clinical efficacy of ICIs; however, their results are inconsistent. In this study, based on updated evidence, we aimed to perform a meta-analysis to clarify the prognostic significance of PPI use in advanced solid cancer patients receiving ICI therapy.

**Methods:**

Eligible literature was searched using PubMed, Cochrane Library, Web of Science, EMBASE, and other network resources before July 2021. Clinical outcome was evaluated using overall survival (OS) and progression-free survival (PFS). The correlation of PPI use with OS or PFS was determined based on hazard ratios (HRs) and 95% confidence intervals (CIs).

**Results:**

A total of 17 studies enrolling 9,978 ICI-treated cancer patients were included in our meta-analysis. The global analysis demonstrated that PPI use was significantly correlated with worse OS [HR = 1.29 (1.10–1.50)] instead of PFS [HR = 1.19 (0.98–1.44)] in solid cancer patients receiving ICI therapy. In a subgroup analysis, the negative correlation of PPI use with ICI efficacy was significant in patients with non-small cell lung cancer [PFS, HR = 1.27 (1.10–1.47)] and urothelial carcinoma [OS, HR = 1.55 (1.31–1.84), PFS, HR = 1.52 (1.13–2.06)] and mixed cohorts containing multiple cancer types [OS, HR = 1.40 (1.16–1.69)], while an opposite result was observed in the PFS of patients with melanoma [HR = 0.48 (0.25–0.90)]. Moreover, the unfavorable prognostic impact of PPI use was also significant in patients over 65 years old [OS, HR = 1.28 (1.05–1.55), PFS, HR = 1.32 (1.12–1.56)] or those receiving anti-PD-1 [OS, HR = 1.37 (1.04–1.79)] or anti-PD-L1 therapies (OS, HR = 1.49 (1.30–1.69), PFS, HR = 1.34 (1.20–1.50). Finally, PPI use was significantly correlated with a worse prognosis in patients receiving PPIs 30 days before and/or after ICI initiation (OS, HR = 1.38 (1.18–1.62), PFS, HR = 1.23 (1.06–1.43)).

**Conclusion:**

Although our global analysis revealed PPI use was not correlated with the PFS of ICI-treated patients, considering the results of our subgroup analysis, PPIs should be still cautiously used shortly before or during ICI therapy. Furthermore, more clinical validations and related mechanism investigations are of great necessity to clarify the clinical correlation of PPI use with ICI efficacy.

**Systematic Review Registration:**

[https://www.crd.york.ac.uk/prospero/], PROSPERO [No. CRD42021243707].

## Introduction

Cancer immunotherapy based on immune checkpoint inhibitors (ICIs) has dramatically revolutionized the current therapeutic strategies of advanced solid tumors. Generally, three representative ICIs have been commonly applied in clinical practice, including programmed cell death 1 (PD-1), programmed cell death ligand 1 (PD-L1), and cytotoxic drug T lymphocyte-associated antigen 4 (CTLA-4) inhibitors (1). However, despite encouraging results from clinical trials and the real world, a fairly large proportion of patients fail to benefit from ICI therapy due to various inherent and environmental factors, such as microsatellite instability, gut microbiota, and concomitant medications (2–4). Previously, our team has demonstrated that antibiotic administration shortly before or after ICI treatment is associated with poor clinical outcomes in patients with advanced solid cancers (5). Moreover, our team has also identified corticosteroid administration for cancer-related indications as an unfavorable prognostic factor in ICI-treated patients (6). Therefore, a further investigation into the correlation between concomitant medications and ICI efficacy will contribute to more individualized management in patients treated with ICI drugs, therefore benefiting their overall prognosis.

Proton pump inhibitors (PPIs) are the most frequently used drugs for the treatment of gastroesophageal reflux disease and peptic ulcers. Functionally, PPIs bind to the H+/K+-ATPases in gastric oxyntic cells to reduce the secretion of gastric acid (7). However, the gut microbiome-based analysis revealed that PPI use may increase the abundance of some potential pathogenic bacteria such as *Escherichia coli*, resulting in an increased risk of enteric infections (8). In cancer epidemiology, recent evidence has indicated that PPI use may be correlated with an increased risk of gastrointestinal malignancies (9, 10). With regard to cancer therapy, on the contrary, PPIs not only function as direct anticancer agents through modifying acidic tumor microenvironment but also enhance the chemosensitivity of cancer cells (11). Therefore, the role of PPIs in the cancer field remains inconclusive, and additional investigations are of great clinical significance.

Recently, emerging studies have found that PPIs may diminish the clinical efficacy of ICIs in patients with advanced solid cancers. For instance, a multicenter retrospective study has demonstrated PPI use was significantly correlated with worse overall survival (OS), progression-free survival (PFS), and lower disease control rate (DCR) and objective response rate (ORR) in patients with locally advanced or metastatic urothelial carcinoma (12). Consistently, another retrospective study based on nationwide data has shown that concomitant PPI use was correlated with reduced OS in non-small cell lung cancer (NSCLC) patients treated with ICIs (13). However, a recent study has found that PPI use was not correlated with ORR, PFS, or OS in advanced renal cell carcinoma (RCC) patients treated with anti-PD-1 therapy alone or plus anti-CTLA-4 therapy (14). Furthermore, researchers have even found that PPI use was significantly correlated with prolonged median PFS in metastatic melanoma patients treated with ICI alone or combined ICIs (15). Considering these inconsistent results, in this study, we performed an up-to-date systematic literature review and meta-analysis to evaluate the prognostic significance of PPI use in ICI-treated patients with advanced solid cancers. Our study will not only contribute to more precise PPI management in clinical practice but also further emphasize the non-negligible correlation of concomitant medications with ICI efficacy.

## Materials and methods

### Literature Search Strategy

**Figure 1:**
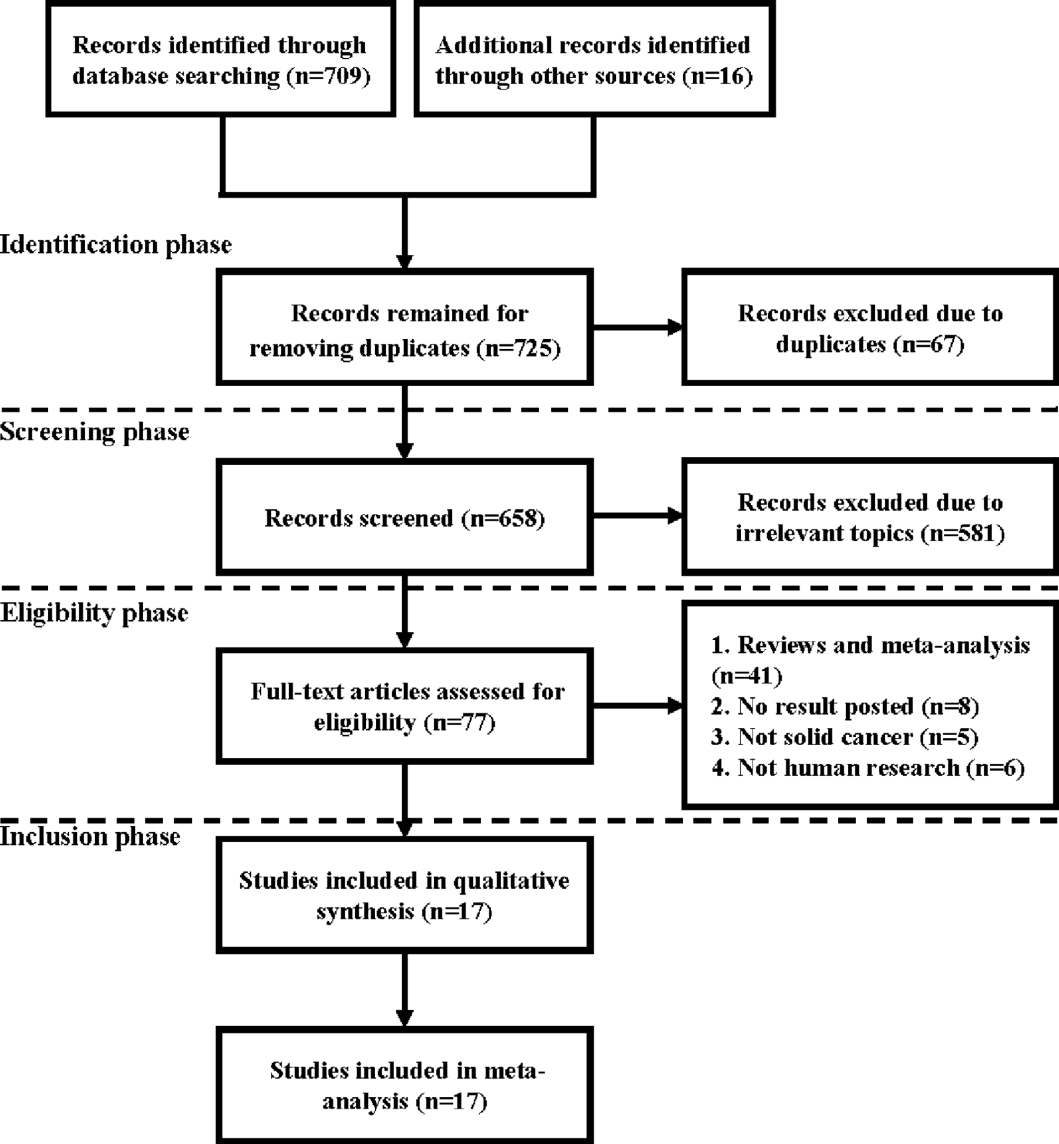
Preferred Reporting Items for Systematic Reviews and Meta-Analyses (PRISMA) flow diagram depicting the literature search process.

The literature search strategy was conducted following the Preferred Reporting Items for Systematic Reviews and Meta-Analyses (PRISMA) guidelines (**Figure 1**). Cochrane Library, PubMed, EMBASE, and Web of Science databases were used to search the related articles investigating the relationship between PPIs and cancer immunotherapy up to July 2021. The search keywords used were as follows: “proton pump inhibitor”, “cancer”, “tumor”, “neoplasm”, “programmed death receptor 1 inhibitor”, “PD-1 inhibitor”, “programmed death-ligand 1 inhibitor”, “PD-L1 inhibitor”, “Immunotherapy”, “cytotoxic T lymphocyte antigen-4 inhibitor”, and “CTLA-4 inhibitor”. The references of research articles and reviews were carefully checked to avoid missing eligible literature. Finally, for including eligible studies as many as possible, we additionally search through the supplementary issues of relevant journals and conference abstracts online.

### Inclusion and Exclusion Criteria

The inclusion criteria are as follows: 1) the study investigating the correlation of PPI use with ICI efficacy in advanced solid cancers; 2) patients treated with ICIs alone or combined with other anticancer therapies; 3) PPIs were used before and/or after ICI initiation; 4) the study containing PPI and non-PPI group; and 5) available prognostic information including OS and/or PFS, hazard ratios (HRs), and 95% CIs. The exclusion criteria are as follows: 1) duplicated studies or data; 2) cellular and/or animal experiments without clinical investigations; 3) reviews and meta-analyses; and 4) studies reported in a language other than English. For duplicated studies, we only included the most informative one in order to ensure data reliability.

### Data Extraction

The following information was collected from the selected articles: publication year, first author, cancer type, region, age, sample size, type of ICI treatment, PPI treatment, PPI exposure, and HRs for OS and PFS. If both univariate and multivariate analyses were available for calculating HRs, the latter was preferred to avoid the impact of confounding factors. The data extraction was performed by two independent researchers, and the inconsistent results were determined by the third researcher.

### Quality Assessment

The Newcastle–Ottawa Quality Assessment Scale (NOS) was used for assessing the quality of included studies. A study that scored no less than 6 points was considered to be of high quality (16). Studies scored less than 6 points were excluded from our meta-analysis, and the exclusion was independently performed by two investigators to avoid bias risk. Any inconsistency was determined by the third researcher.

### Statistical Analysis

The present analyses were performed using Stata SE14.0. The heterogeneity of the included studies was evaluated using Cochrane’s Q test and I^2^ statistics. For low-heterogeneity studies (p > 0.05 and I^2^ ≤ 50%), a fixed-effects model was used for analysis. While for high heterogeneity (p < 0.05 or I^2^ > 50%), a random-effects model was used for analysis. The stability of the results was assessed using sensitivity analysis, and publication bias was assessed using Begg’s test and Egger’s test. An observed HR > 1 indicated that PPI use was negatively correlated with OS or PFS. For all the statistical analyses, a p-value less than 0.05 was considered to be statistically significant.

## Results

### Study Selection

The flowchart of study selection is shown in **Figure 1** *. A* total of 709 related literature were identified from electronic databases, with 16 additional articles from other sources. After duplicate studies (n = 67) and irrelevant topics (n = 581) were removed, 77 literature were reserved. Next, the remaining studies were further screened by excluding reviews or meta-analyses (n = 41), studies with unavailable results (n = 8), studies unrelated with solid tumors (n = 5), and non-clinical studies (n = 6). Finally, a total of 17 articles published between January 2016 and July 2021 were included in our meta-analysis [(12, 15, 17–31)].

### Characteristics of the Included Studies

The characteristics of 17 studies are summarized in **Table 1**. *A* total of 9,978 patients who received anti-PD-(L)1 and/or anti-CTLA-4 therapy were enrolled in our meta-analysis. Six studies were from the United States, while five and three were from Europe and Asia, respectively. The most commonly diagnosed cancer was NSCLC, followed by melanoma and urothelial carcinoma. The majority of patients received PPI treatment shortly before and/or after ICI initiation, and omeprazole was the most commonly used PPI drug. As shown in **Table 2**, all the included studies were retrospective investigations with available prognostic information (OS and/or PFS). HR values were extracted from the multivariate analysis from 11 studies and the univariate analysis from 3 studies. The NOS scoring system indicated that all the included studies were scored no less than 6 points, suggesting the high quality of data sources.

**Table 1:** Baseline characteristics of the included studies.

Author	Year	Age	Region	Cancer type	ICI treatment	PPI treatment	No. of PPI	Patients	PPI exposure
Afzal et al. (15)	2019	65	America	Melanoma	PD-1, CTLA-4	Omeprazole (majority)	29	120	Within
Chalabi et al. (19)	2020	NA	Worldwide	NSCLC	PD-L1	Omeprazole, pantoprazole, lansoprazole, rabeprazole, esomeprazole, dexlansoprazole	234	757	Prior, within (30 days)
Failing et al. (21)	2016	58	America	Melanoma	CTLA-4	Omeprazole (majority)	17	80	Within
Hakozaki et al. (22)	2019	67	Asia	NSCLC	PD-1	NA	47	90	Prior (30 days)
Iglesias‐Santamaría (25)	2019	66	Europe	Multiple	ICI	NA	78	102	Within
Svaton et al. (30)	2020	67	Europe	NSCLC	PD-1	Omeprazole, pantoprazole, lansoprazole	64	224	Prior, Within (30 days)
Zhao et al. (31)	2019	62	Asia	NSCLC	PD-1, other	NA	40	109	Prior, Within (30 days)
Hopkins et al. (23)	2020	68	Worldwide	UC	PD-L1	Meprazole, pantoprazole, lansoprazole, rabeprazole, esomeprazole, dexlansoprazole	471	1360	Prior, Within (30 days)
Cortellini et al. (20)	2020	69	Europe	Multiple	PD-(L)1	NA	491	1012	Within
Husain et al. (24)	2021	62	America	Multiple	ICI	NA	415	1091	Within
Stokes et al (29).	2021	69	America	NSCLC	PD-(L)1	Omeprazole (majority)	2159	3634	within (90 days)
Peng et al. (28)	2021	64	America	Multiple	PD-1, CTLA-4	NA	89	233	Prior, Within (30 days)
Jun et al. (26)	2021	66	Worldwide	HCC	PD-1, CTLA-4, TKI	Omeprazole, pantoprazole, lansoprazole, rabeprazole, esomeprazole, dexlansoprazole	85	314	Prior (30 days)
Ruiz-Bañobre et al. (12)	2021	69	Europe	UC	PD-(L)1	NA	54	119	Prior (30 days)
Araujo et al. (17)	2021	59	America	Multiple	ICI	NA	57	216	Prior (60 days)
Miura et al. (27)	2021	65	Asia	NSCLC	PD-1	Lansoprazole, rabeprazole Esomeprazole	163	300	Within
Buti et al. (18)	2021	69	Europe	Multiple	ICI	NA	104	217	Within

NSCLC, non-small cell lung cancer; PPI, proton pump inhibitor; UC, urothelial carcinoma; HCC, hepatocellular carcinoma; NA, not available; ICI, immune checkpoint inhibitor; CTLA-4, cytotoxic T-lymphocyte-associated antigen 4; PD-1, programmed cell death protein-1; PD-L1, programmed cell death ligand 1.

**Table 2:** Prognostic information and quality assessment of the included studies.

Author	Year	Method	Outcome	HR (95% CI)	HR (95% CI)	Analysis	NOS score
				for OS	for PFS		
Afzal et al. (15)	2019	RE	OS/PFS	1.01 (0.40–2.00)	0.30 (0.10–0.70)	M	7
Chalabi et al. (19)	2020	RE	OS/PFS	1.45 (1.20–1.75)	1.30 (1.10–1.53)	NA	8
Failing et al. (21)	2016	RE	OS/PFS	0.44 (0.17–1.15)	0.60 (0.34–1.06)	NA	7
Hakozaki et al. (22)	2019	RE	OS	1.90 (0.80–4.51)	NA	M	6
Iglesias‐Santamaría (25)	2019	RE	OS/PFS	0.79 (0.40–1.56)	0.75 (0.42–1.34)	M	7
Svaton et al. (30)	2020	RE	OS/PFS	1.22 (0.72–2.05)	1.36 (0.89–2.06)	M	8
Zhao et al. (31)	2019	RE	OS/PFS	0.68 (0.33–1.43)	0.91 (0.54–1.54)	U	8
Hopkins et al. (23)	2020	RE	OS/PFS	1.52 (1.27–1.83)	1.38 (1.18–1.62)	M	8
Husain et al. (24)	2021	RE	OS	1.99 (1.15–3.45)	NA	NA	7
Stokes et al. (29)	2021	RE	OS	0.96 (0.89–1.04)	NA	M	7
Peng et al. (28)	2021	RE	OS/PFS	1.20 (0.77–1.87)	0.90 (0.64–1.28)	M	8
Jun et al. (26)	2021	RE	OS	1.14 (0.84–1.54)	NA	U	6
Ruiz-Bañobre et al. (12)	2021	RE	OS/PFS	1.83 (1.11–3.02)	1.94 (1.22–3.09)	M	8
Araujo et al. (17)	2021	RE	OS/PFS	1.73 (1.23–2.44)	2.36 (1.67–3.34)	M	8
Miura et al. (27)	2021	RE	OS	1.36 (0.96–1.91)	NA	M	7
Cortellini et al. (20)	2020	RE	OS/PFS	1.26 (1.04–1.52)	1.26 (1.07–1.48)	M	8
Buti et al. (18)	2021	RE	OS	1.57 (1.13–2.18)	NA	U	6

RE, retrospective; OS, overall survival; PFS, progression-free survival; HR, hazard ratio, NA, not available; U, univariate; M, multivariate; NOS, Newcastle–Ottawa Quality Assessment Scale.

### Clinical Correlation of Proton Pump Inhibitor Use With Overall Survival and Progression-Free Survival

**Figure 2:**
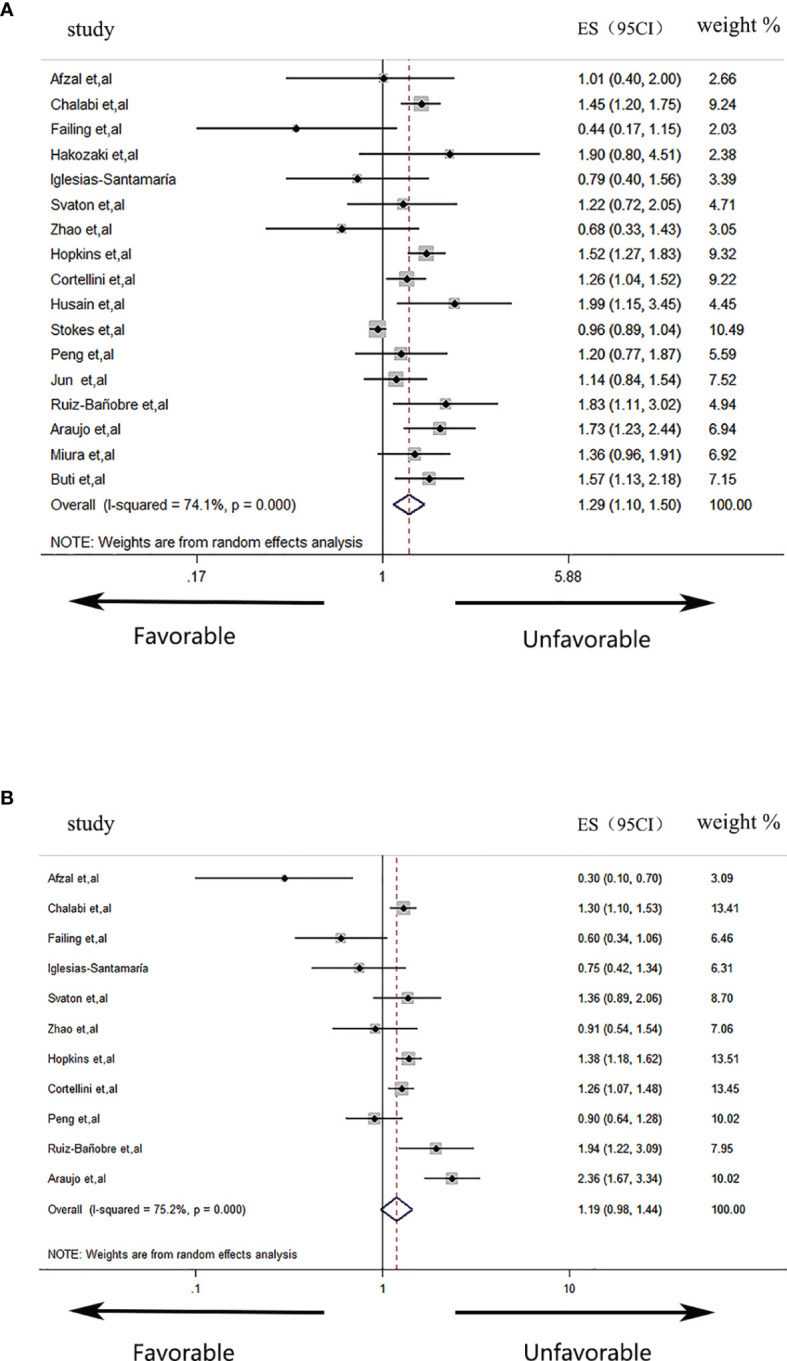
Forest plots of the hazard ratios (HRs) for the correlations of proton pump inhibitor (PPI) use with overall survival **(A)** and progression-free survival **(B)**.

As shown in **Figure 2A**, the pooled meta-analysis for OS included 17 studies with 9,978 solid cancer patients. With the use of a random-effects model (I^2^ = 74.1%, p < 0.001), the result indicated that concomitant PPI use was significantly correlated with worse OS in patients treated with ICI drugs (HR = 1.29, 95% CI: 1.10–1.50). As shown in **Figure 2B**, the pooled meta-analysis for PFS included 11 studies with 4,332 solid cancer patients. However, using the same model (I^2^ = 75.2%, p < 0.001), the result suggested that concomitant PPI use was not significantly correlated with the PFS of patients treated with ICI drugs (HR = 1.19, 95% CI: 0.98–1.44).

### Subgroup Analyses for the Prognostic Significance of Proton Pump Inhibitor Use

To further evaluate the prognostic impact of PPI use, a subgroup analysis was performed based on region, cancer type, age, sample size, ICI therapy, and PPI exposure timeframe. The analysis results are summarized in **Tables 3**, **4**. In the region subgroup, PPI use was significantly correlated with worse OS in patients from worldwide multicenter studies (HR = 1.41 (1.23–1.63)) and Europe (HR = 1.34 (1.11–1.62)), while its correlation with worse PFS was only significant in patients from worldwide multicenter studies (HR = 1.34 (1.20–1.50)). In cancer types, PPI use was significantly correlated with worse PFS in patients with NSCLC (HR = 1.27 (1.10–1.47)) or urothelial carcinoma (HR = 1.52 (1.13–2.06)), while the opposite result was observed in patients with melanoma (HR = 0.48 (0.25–0.90)). In terms of age, the negative prognostic impact of PPI use was only significant in patients older than 65 years (OS, HR = 1.28 (1.05–1.55), PFS, HR = 1.32 (1.12–1.56)). With regard to sample size, its negative prognostic impact was only significant in studies with a sample size of more than 200 (OS, HR = 1.34 (1.14–1.58), PFS, HR = 1.35 (1.14–1.60)). In the subgroup analysis stratified by ICI therapies, PPI use was significantly correlated with worse OS in patients treated with anti-PD-1 (HR = 1.37 (1.04–1.79)), anti-PD-L1 (HR = 1.49 (1.30–1.69)), and ICI therapies (HR = 1.54 (1.16–2.05)). However, this negative correlation with PFS was only significant in patients treated with anti-PD-L1 therapies (HR = 1.34 (1.20–1.50)). Finally, the correlation between PPI exposure timeframes and ICI efficacy was investigated. The result demonstrated that the negative correlation of PPI use with OS was significant in patients receiving PPI drugs at any time after ICI initiation (∞, HR = 1.27 (1.01–1.59)) and 30 days before and/or after ICI initiation ( ± 30, HR = 1.38 (1.18–1.62); −30, HR = 1.43 (1.00–2.05)). Moreover, this correlation with PFS was significant in patients receiving PPI drugs 30 days before and/or after ICI initiation ( ± 30, HR = 1.23 (1.06–1.43)).

**Table 3:** Subgroup analysis for the correlation of proton pump inhibitor use with overall survival.

Subgroup	No. of studies	OS hazard ratios (95% CI)	p-Value	Heterogeneity	
				I^2^	p-Value
Region					
Asia	3	1.21 (0.74–1.98)	0.439	47.00%	0.152
America	6	1.19 (0.85–1.67)	0.305	75.50%	0.001
Europe	5	1.34 (1.11–1.62)	0.003	23.20%	0.267
Worldwide	3	1.41 (1.23–1.63)	<0.001	22.90%	0.273
Cancer type					
NSCLC	6	1.19 (0.92–1.54)	0.180	77.10%	0.001
Melanoma	2	0.70 (0.31–1.56)	0.379	41.10%	0.192
Urothelial carcinoma	2	1.55 (1.31–1.84)	<0.001	0%	0.495
Multiple	6	1.40 (1.16–1.69)	<0.001	36.00%	0.167
Age group					
≤65	7	1.23 (0.92–1.66)	0.169	55.00%	0.038
>65	9	1.28 (1.05–1.55)	0.014	78.20%	<0.001
Sample size					
≤200	6	1.01 (0.64–1.60)	0.954	57.10%	0.040
>200	11	1.34 (1.14–1.58)	<0.001	80.00%	<0.001
PPI exposure					
∞	7	1.27 (1.01–1.59)	0.038	46.00%	0.085
± 30	5	1.38 (1.18–1.62)	<0.001	25.00%	0.255
−30	3	1.43 (1.00–2.05)	0.052	37.70%	0.201
Immunotherapy drug					
PD-1, CTLA-4	2	1.15 (0.78–1.70)	0.473	0%	0.713
PD-L1	2	1.49 (1.30–1.69)	<0.001	0%	0.725
PD-1	3	1.37 (1.04–1.79)	0.025	0%	0.691
ICI	4	1.54 (1.16–2.05)	0.003	39.20%	0.177
PD-(L)1	3	1.20 (0.90–1.61)	0.212	83.70%	0.002

NSCLC, non-small cell lung cancer; HR, hazard ratio; OS, overall survival; PD-1, programmed cell death protein-1; PD-L1, programmed cell death ligand 1.

**Table 4:** Subgroup analysis for the association of proton pump inhibitor use with progression-free survival.

Subgroup	No. of studies	PFS hazard ratios (95% CI)	p-Value	Heterogeneity	
				I^2^	p-Value
Region					
America	4	0.85 (0.39–1.84)	0.683	90.2%	<0.001
Europe	4	1.29 (0.98–1.70)	0.068	53.5%	0.091
Worldwide	2	1.34 (1.20–1.50)	<0.001	0%	0.609
Cancer type					
NSCLC	3	1.27 (1.10–1.47)	0.001	0%	0.420
Melanoma	2	0.48 (0.25–0.90)	0.023	31.2%	0.228
Urothelial carcinoma	2	1.52 (1.13–2.06)	0.006	45.9%	0.174
Multiple	4	1.23 (0.81–1.85)	0.326	84.4%	<0.001
Age					
≤65	5	0.88 (0.48–1.61)	0.670	87.3%	<0.001
>65	5	1.32 (1.12–1.56)	0.001	42.2%	0.140
Sample size					
≤200	5	0.81 (0.46–1.41)	0.450	77.3%	0.001
>200	6	1.35 (1.14–1.60)	<0.001	69.0%	0.007
PPI exposure					
∞	4	0.72 (0.40–1.28)	0.259	80.70%	0.001
± 30	5	1.23 (1.06–1.43)	0.007	39.10%	0.160
Immunotherapy drug					
PD-1, CTLA-4	2	0.57 (0.20–1.65)	0.303	77.00%	0.037
PD-L1	2	1.34 (1.20–1.50)	<0.001	0%	0.609
ICI	2	1.36 (0.44–4.19)	0.588	91.00%	0.001
PD-(L)1	2	1.48 (0.98–2.22)	0.061	66.10%	0.086

NSCLC, non-small cell lung cancer; HR, hazard ratio; PFS, progression-free survival; PD-1, programmed cell death protein-1; PD-L1, programmed cell death ligand 1.

### Publication Bias and Sensitivity Analysis

**Figure 3:**
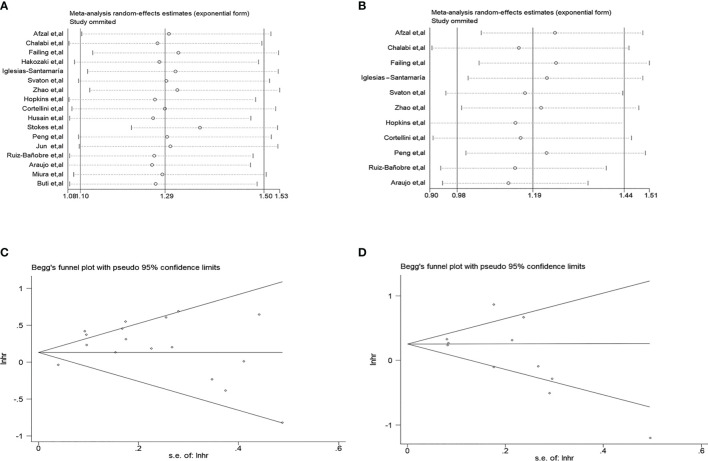
Sensitivity analysis and publication bias. **(A)** Sensitivity analysis for the hazard ratios (HRs) of overall survival (OS). **(B)** Sensitivity analysis for the HRs of progression-free survival (PFS). **(C)** Begg’s funnel plots for assessing the publication bias of OS. **(D)** Begg’s funnel plots for assessing the publication bias of PFS.

The sensitivity analysis was performed to evaluate the bias caused by the limited included literature (**Figures 3A, B**). The results indicated that no single study could significantly affect the pooled HRs of OS and PFS, suggesting the result’s reliability. Moreover, Begg’s (**Figures 3C, D**) and Egger’s tests were used to assess publication bias. The result demonstrated that no significant publication bias existed in calculating the HR values of OS (Begg’s test, p = 0.077; Egger’s test, p = 0.110) and PFS (Begg’s test, p = 0.072; Egger’s test, p = 0.197).

## Discussion

Despite the recognition for ICI-based cancer immunotherapies by the 2018 Nobel Prize, in clinical practice, the majority of patients fail to benefit from these therapies, even in combined therapies with two ICI drugs and/or chemoradiotherapy (32). A recent review has proposed that the anticancer efficacy of ICI drugs may be affected by some concomitant medications such as antibiotics, corticosteroids, PPIs, and metformin (3). For instance, antibiotic administration was proved to diminish the clinical efficacy of ICI drugs by influencing the gut microbiome (33). Similar to antibiotics, PPIs were also found to significantly affect the composition of the gut microbiota and are negatively associated with ICI efficacy (23, 34, 35). However, there are some studies suggesting that PPI use was unrelated to ICI efficacy (14, 28). Therefore, in this study, we performed an up-to-date meta-analysis to clarify the correlation between PPI use and ICI efficacy in advanced solid cancers. During the preparation of our manuscript, it is worth mentioning that three similar studies have been published so far (36–38). Compared with them, on the one hand, our present work is an up-to-date analysis including more studies (n = 17 vs. n = 7 for Li et al., n = 5 for Li et al., and n = 7 for Qin et al.) and more patients (n = 9,978 vs. n = 1,482 for Li et al., n = 1,167 for Li et al., and n = 3,647 for Qin et al.), especially including 8 studies published in 2021, which have not been included in these three studies. On the other hand, in addition to cancer types, our subgroup analysis was performed based on more factors including region, age, sample size, therapeutic strategy, and PPI exposure timeframes, contributing to further understanding of the actual role of PPIs during ICI therapy. Moreover, the results varied wildly among these three meta-analyses. Therefore, our up-to-date investigation is of great necessity and hoped to provide novel insights into the precise management of PPIs in clinical practice.

In our study, we firstly found that PPI use was significantly correlated with worse OS in patients treated with ICI therapy, while no significant correlation was found between it and PFS. The two meta-analyses published in 2020 have collectively demonstrated that PPI use was unrelated to OS nor PFS of patients treated with ICI drugs (36, 37). Both the studies have attributed their results to the sophisticated role of PPIs in cancer therapy. For instance, PPIs may disturb the gut microbiome that was related to ICI efficacy and was suggested to be discontinued or switched to a histamine H2-receptor antagonist (39). However, accumulating evidence demonstrated PPIs not only inhibit tumor growth and enhance chemosensitivity through modulating an acid milieu but also promote immune reaction and prevent tumor immune escape (40). Furthermore, compared with antibiotics, the impact of PPIs on the gut microbiome is relatively limited, and there is a lack of sequencing studies to identify dominant bacteria affected by PPIs in ICI non-responders. On the contrary, an updated investigation by Qin et al. in 2021 demonstrated a significant negative correlation of PPI use with OS and PFS in advanced cancer patients treated with ICIs (38). Despite the lack of supporting mechanism investigations, Qin et al. hypothesized that PPIs may decrease ICI efficacy by altering the abundance of some bacteria *via* increasing pH levels. Our present result revealed that PPI use was unrelated to ICI efficacy, which is consistent with 5 of 11 included studies (21, 25, 28, 30, 31). Despite the fact that PFS is superior to OS in evaluating therapy response, however, we were unable to assert that PPI use was unrelated to PFS. In the future, multicenter validations based on large samples are still essential, and cautious use of PPIs for ICI-treated patients is advocated.

To further investigate the correlation of PPI with ICI efficacy, the subgroup was stratified according to region, cancer type, sample size, the timeframe of PPI exposure, and immunotherapy drug. In region subgroups, PPI use was significantly correlated with worse OS and PFS of patients from worldwide multicenter studies, but the number of included studies is limited (n = 2–3), and more cross-continent multicenter cooperation is encouraged. In cancer-type subgroups, the negative correlation of PPI use with PFS was significant in patients with NSCLC and urothelial carcinoma, while it was the opposite in patients with melanoma. Metagenomic evidence has proved Ruminococcaceae was enriched in the stools of NSCLC patients benefiting from ICIs, while PPIs were known to be negative with Ruminococcaceae abundance (4, 41). Therefore, PPIs may diminish the clinical efficacy of ICIs partly through modulating gut microbiota in NSCLC. A previous study has found esomeprazole as a PPI that inhibited the malignant progression of melanoma through modifying the acidic microenvironment (42). A similar study also found that esomeprazole inhibited the growth of melanoma through downregulating VEGF-C expression *via* inactivating NF-κB (43). Both the studies supported the hypothesis that PPIs may directly exert their anticancer role rather than affecting ICI efficacy in melanoma, partly explaining the opposite results in the subgroup analysis. In age subgroups, PPI use was significantly correlated with worse OS and PFS only in patients over 65 years old. Considering the fact that the elderly received PPI therapy more frequently than other age groups, PPIs should be especially cautiously used or switched to histamine H2-receptor antagonists in these patents during immunotherapy. In terms of sample size, we found that the negative correlation of PPIs with OS/PFS was significant in studies enrolling more than 200 patients, although the included studies were limited. This finding highlights the importance of sample size in determining the prognostic role of PPIs and encourages large-scale clinical validations in the future. Since the timeframes of PPI exposure varied among studies, the subgroup analysis was subsequently performed to clarify whether the unfavorable prognostic impact of PPIs was dependent on their exposure timeframes (18, 19, 22). As result, we found that the negative correlation of PPIs with OS and PFS was significant in patients receiving PPIs 30 days before and/or after ICI initiation. This finding suggests that PPIs should be discontinued shortly before or after starting ICI therapy in order to avoid their potential unfavorable impact. Finally, we noted the unfavorable prognostic impact of PPIs was significant in patients receiving anti-PD-L1 therapies. However, considering limited included studies in each subgroup, more retrospective investigations are needed, which may help further clarify the correlation between PPIs and the efficacy of various types of ICIs.

There are several limitations to our study. First, this is a meta-analysis based on previously published retrospective studies. During data extraction, much important information such as HR values of OS/PFS, PPI types, PPI exposure timeframes, and ICI therapeutic strategies was unavailable or not detailed. This limitation partly impedes our global and subgroup analyses and is hoped to be improved by following up-to-date investigations based on more high-quality literatures. Second, the included studies were restricted to literatures published in English, which may miss some potential eligible ones published in other languages such as Chinese. In the future, international collaborations are highly encouraged and will benefit from a more comprehensive literature search. Third, various inherent factors of included retrospective studies such as patient selection, drug types/doses, and therapeutic strategies may also impact our result. Fourth, our subgroup analysis mainly focused on NSCLC, melanoma, and urothelial carcinoma due to limited studies. The correlation of PPIs with ICI efficacy in other cancers such as digestive malignancies is worth investigating. Fifth, since our finding is partially inconsistent with other similar studies, one or more independent cohorts from our hospital should be utilized for further validation. Finally, the direct evidence supporting the correlation between PPI use and ICI efficacy is still insufficient. For instance, to our knowledge, there are no studies investigating the changes of the gut microbiome in NSCLC patients receiving PPIs during ICI therapy, nor the experiments validating the beneficial role of PPIs in enhancing the anticancer efficacy of ICI drugs in melanoma. Therefore, mechanism investigations based on multi-omics detection and animal experiment will be emphasized in our following work.

## Conclusion

In conclusion, based on updated evidence, our study demonstrated that concomitant PPI was significantly correlated with worse OS instead of PFS in advanced solid cancer patients receiving ICIs. The subgroup analysis revealed the negative correlation of PPI use with PFS was significant in NSCLC and urothelial carcinoma, while it was the opposite for melanoma. Furthermore, the unfavorable prognostic impact of PPIs was observed in patients receiving anti-PD-L1/PD-1 therapies or those receiving PPIs 30 days before and/or after ICI initiation. Therefore, although our global analysis failed to confirm the negative correlation of PPIs with PFS, cautious use of PPIs is still recommended to avoid their potentially detrimental impact on ICI efficacy. In addition, more clinical validations and related up-to-date meta-analyses are necessary, and mechanism investigations *in vivo* and *in vitro* will be helpful in elucidating the complicated role of PPIs in cancer immunotherapy.

## Data Availability Statement

The original contributions presented in the study are included in the article/supplementary material. Further inquiries can be directed to the corresponding authors.

## Author Contributions

XY and MY: conceptualization and methodology. CL and HG: data collection and analysis and writing—original draft preparation. HM and JT: data validation and writing—reviewing and editing.

## Funding

This study was supported by the Traditional Chinese Medicine Science and Technology Development Plan Project of Jiangsu (No. YB2020107) and Yangzhou Science and Technology Bureau (No. YZ2020085).

## Conflict of Interest

The authors declare that the research was conducted in the absence of any commercial or financial relationships that could be construed as a potential conflict of interest.

## Publisher’s Note

All claims expressed in this article are solely those of the authors and do not necessarily represent those of their affiliated organizations, or those of the publisher, the editors and the reviewers. Any product that may be evaluated in this article, or claim that may be made by its manufacturer, is not guaranteed or endorsed by the publisher.
